# General intelligence in adult patients with early- and adult-onset schizophrenia

**DOI:** 10.1192/j.eurpsy.2023.1048

**Published:** 2023-07-19

**Authors:** T. Calkova, L. Mørch-Johnsen, R. Elle Smelror, K. Nordbø Jørgensen, S. Cervenka, K. Collste, A. Vaskinn, A. Margrethe Myhre, O. A. Andreassen, T. Ueland, I. Agartz, D. Andreou

**Affiliations:** 1 Centre for Clinical Research, Västmanland County Hospital Västerås, Uppsala University, Västerås; 2 Centre for Psychiatry Research, Department of Clinical Neuroscience, Karolinska Institutet & Stockholm Health Care Services, Stockholm, Sweden; 3 Norwegian Centre for Mental Disorders Research (NORMENT), Institute of Clinical Medicine, University of Oslo, Oslo; 4 Department of Psychiatry & Department of Clinical Research, Østfold Hospital, Grålum; 5 Department of Psychiatric Research, Diakonhjemmet Hospital, Oslo; 6 Department of Psychiatry, Telemark Hospital, Skien, Norway; 7 Department of Medical Sciences, Psychiatry, Uppsala University, Uppsala; 8 Centre for Research and Education in Forensic Psychiatry, Oslo University Hospital, Oslo, Sweden; 9 Norwegian Centre for Mental Disorders Research (NORMENT), Division of Mental Health and Addiction; 10 Psychosis Research Section, Oslo University Hospital; 11 Department of Psychology, University of Oslo, Oslo, Norway

## Abstract

**Introduction:**

Early-onset schizophrenia (EOS) is a relatively uncommon disorder with psychotic symptoms emerging before 18 years of age. Although still under debate, EOS may be a more severe disorder relative to adult-onset schizophrenia (AOS), with worse prognosis. Cognitive deficits are a core feature of schizophrenia, accounting for a large part of the detrimental effect of the disorder and may reflect underlying neurodevelopmental disturbances. Some but not all previous studies show that the magnitude of cognitive deficits, including intelligence quotient (IQ), in patients with schizophrenia is dependent on the age of onset.

**Objectives:**

We aimed to assess IQ in adult patients with EOS and AOS, and healthy controls. We hypothesized that patients with EOS would show lower IQ than those with AOS, and both patient groups lower IQ than HC.

**Methods:**

We included 136 adult patients with EOS (mean age: 24.7 (7.7) years, mean duration of illness: 9.3 (8.5) years, 50% women), 382 patients with AOS (mean age: 32.4 (9.5) years, mean duration of illness: 5.7 (6.6) years, 40.1% women) and 896 adult healthy controls (mean age: 33.2 (9.2) years, 47.1% women). We assessed current IQ with the Wechsler Abbreviated Scale of Intelligence (WASI) which yielded verbal (VIQ), performance (PIQ) and full-scale IQ (FIQ) scores. In a post-hoc analysis, we estimated premorbid IQ using the National Adult Reading Test (NART). We applied analyses of covariance (ANCOVAs) to investigate the putative differences in IQ scores and IQ change between patients with EOS, patients with AOS and healthy controls.

**Results:**

In sex-, and age-adjusted models, FIQ and PIQ, but not VIQ, were significantly lower in EOS than in AOS (p=0.03, p<0.001 and p=0.428, respectively) (Image). Patients with EOS had fewer years of education than patients with AOS (p<0.001); the PIQ but not the FIQ difference between EOS and AOS remained significant after adjustment for education years (p=0.016 and p=0.333, respectively). Both patient groups had significantly lower IQ scores than healthy controls (Image). Further, patients with EOS and patients with AOS did not significantly differ in estimated premorbid IQ (109 and 110 units, respectively, p=0.092), whereas patients with EOS had a significantly larger estimated IQ decline after the disease onset compared to patients with AOS (12 and 9 units decline, respectively, p=0.015).

**Image:**

**Figure:**
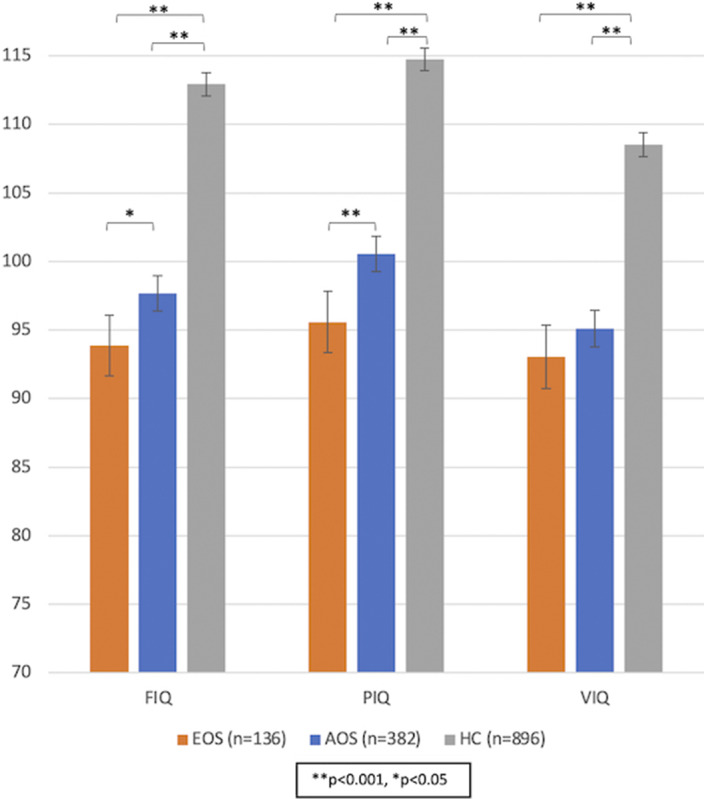

**Conclusions:**

Our findings show that adult patients with EOS have significantly lower PIQ and FIQ scores, and significantly larger IQ decline after the disease onset, but not lower premorbid IQ, compared to patients with AOS. The adolescent onset of psychotic symptoms is linked, as expected, to fewer total years of education, which appears to explain the lower FIQ but only partially the lower PIQ in EOS, which may thereby be linked to the disorder per se.

**Disclosure of Interest:**

T. Calkova: None Declared, L. Mørch-Johnsen: None Declared, R. Elle Smelror: None Declared, K. Nordbø Jørgensen: None Declared, S. Cervenka: None Declared, K. Collste: None Declared, A. Vaskinn: None Declared, A. Margrethe Myhre: None Declared, O. A. Andreassen Consultant of: HealthLytix, Speakers bureau of: Lundbeck and Sunovion, T. Ueland: None Declared, I. Agartz: None Declared, D. Andreou: None Declared

